# Survival and critical care use among people with dementia in a large English cohort

**DOI:** 10.1093/ageing/afad157

**Published:** 2023-09-08

**Authors:** Emel Yorganci, Katherine E Sleeman, Elizabeth L Sampson, Robert Stewart, The EMBED-Care Programme, Elizabeth L Sampson, Catherine J Evans, Katherine E Sleeman, Nuriye Kupeli, Kirsten J Moore, Nathan Davies, Clare Ellis-Smith, Jane Ward, Anna Gola, Bridget Candy, Rumana Z Omar, Jason D Warren, Janet E Anderson, Richard Harding, Robert Stewart, Simon Mead

**Affiliations:** Cicely Saunders Institute of Palliative Care, Policy & Rehabilitation, King's College London, Florence Nightingale Faculty of Nursing, Midwifery & Palliative Care, London SE5 9PJ, UK; Cicely Saunders Institute of Palliative Care, Policy & Rehabilitation, King's College London, Florence Nightingale Faculty of Nursing, Midwifery & Palliative Care, London SE5 9PJ, UK; Division of Psychiatry, University College London, London W1T 7NF, UK; Liaison Psychiatry, Royal London Hospital, East London NHS Foundation Trust, London E1 1FR, UK; Department of Psychological Medicine, Institute of Psychiatry, Psychology and Neuroscience, King's College London, London SE5 8AB, UK; South London and Maudsley NHS Foundation Trust, London SE5 8AB, UK

**Keywords:** critical care, dementia, survival, routine data, intensive care, older people

## Abstract

**Background:**

Admitting people with dementia to critical care units may not always lead to a clear survival benefit. Critical care admissions of people with dementia vary across countries. Little is known about the use and trends of critical care admissions of people with dementia in England.

**Objective:**

To investigate critical care use and survival among people with dementia in a large London catchment area.

**Methods:**

A retrospective cohort study using data from dementia assessment services in south London, UK (2007–20) linked with national hospitalisation data to ascertain critical care admissions. Outcomes included age–sex-standardised critical care use and 1-year post-critical care admission survival by dementia severity (binary: mild versus moderate/severe). We used logistic regression and Kaplan–Meier survival plots for investigating 1-year survival following a critical care admission and linear regressions for time trends.

**Results:**

Of 19,787 people diagnosed with dementia, 726 (3.7%) had ≥1 critical care admission at any time after receiving their dementia diagnosis. The overall 1-year survival of people with dementia, who had a CCA, was 47.5% (*n* = 345). Dementia severity was not associated with 1-year survival following a critical care admission (mild dementia versus moderate–severe dementia odds of 1-year mortality OR: 0.90, 95% CI [0.66–1.22]). Over the 12-year period from 2008 to 2019, overall critical care use decreased (*β* = −0.05; 95% CI = −0.01, −0.0003; *P* = 0.03), while critical care admissions occurring during the last year of life increased (*β* = 0.11, 95% CI = 0.01, 0.20, *P* = 0.03).

**Conclusions:**

In this cohort, while critical care use among people with dementia declined overall, its use increased among those in their last year of life. Survival remains comparable to that observed in general older populations.

## Key Points

- In a large cohort with a dementia diagnosis, while overall critical care use decreased over the years, a slight increase was observed among critical care admissions which took place in the last year of the decedents’ lives.
- One-year survival of people with dementia following a critical care admission was 48%, similar to that observed in general older populations.
- Data linkages and clinically relevant routinely collected data are essential for informing service planning and evaluating the care quality of people with dementia.

## Introduction

The number of people living with dementia is increasing [1], and providing high-quality and equitable care is a major challenge. Understanding different aspects of health care use is important for service planning, including critical care unit admissions [2]. While critical care admissions (CCAs) may be life-saving, those for people with dementia need careful consideration as to whether benefits outweigh burdens [3] and whether they are in accord with people’s previous preferences, especially at advanced stages of dementia [4]. CCAs in dementia may vary geographically and over time; those in the last year of life have increased in several countries [4, 5], but trends in England have not been described. We investigated CCAs among people with dementia from the point of diagnosis, including survival and time trends, by leveraging a novel data linkage in a large south London catchment area.

## Methods

### Setting and data sources

A retrospective cohort was constructed using data linkages between (i) dementia assessment/management services (South London and Maudsley [SLaM] National Health Service Foundation Trust Clinical Record Interactive Search [CRIS]), (ii) hospital inpatient (2008–March 2020), (iii) hospital critical care (2008–March 2020), and (iv) the national death registry (Office of National Statistics [ONS]). Mortality information was available up to March 2021 to allow at least 1 year of follow-up. The source database is approved for secondary analysis (Oxfordshire Research Ethics Committee C, reference 18/SC/0372). Hospital Episode Statistics (HES) Adult Inpatient Care contains details of all admissions in England, and HES Adult Critical Care contains details of CCAs to intensive care units and high dependency units [2]. SLaM provides mental health services to a catchment population of 1.2 million south London residents; the CRIS platform provides research access to de-identified structured and open-text data from SLaM’s electronic health records [6], including its comprehensive dementia assessment services.

We included any person with dementia who was ≥50 years of age at diagnosis between 1 January 2006 and 31 March 2020. All dementia diagnoses recorded in CRIS were determined from ICD-10 diagnosis codes (F00x–03x) recorded in structured fields.

### Clinical variables

We used Mini Mental State Examination (MMSE [7]) scores recorded on the closest date to the CCA to estimate dementia severity, applying recommended cut-off points [8] (mild = MMSE ≥ 20, moderate–severe = MMSE <20). The MMSE has good test–retest reliability and acceptable sensitivity and specificity to detect mild-to-moderate stages of dementia [9, 10]. For people with no recorded MMSE score, the cognitive problems score from the Health of the Nation Outcome Scales [11], a structured clinical outcome measure used routinely in English mental health care, was used to estimate dementia severity with standard recommended cut-off points (mild = 0–2; moderate–severe = 3–4 [11]). For each participant, we retrieved information on CCAs after the date of their first CRIS-recorded dementia diagnosis, and we linked the information on CCAs to information from HES Admitted Patient Care to capture the length of critical care unit and overall hospital stay. For each person, we had dates of hospital admission, critical care unit admission, critical care unit discharge and hospital discharge.

### Outcomes

Outcomes included 1-year survival following CCA by dementia severity and CCA use. One-year survival was determined by checking whether the individual who had CCA was living or not 365 days after their discharge from the critical care unit. We reported the percentage of people with dementia who had a CCA and the CCA rate (the number of CCAs divided by the person-years of follow-up of the overall cohort). We calculated age–sex-standardised annual critical care use (number of CCAs of people with dementia per year/number of people living with dementia per year). For standardisation, we used England’s mid-year population estimates to adjust the number of CCAs observed in our sample for sex and age groups (50–59, 60–69, 70–79, 80–89 and >90) [12]. We calculated age–sex-standardised critical care use in the last year of life (number of CCAs of people with dementia in the last year of life per year/number of decedents with dementia per year). For standardisation, we used England’s leading causes of death statistics to identify the annual dementia deaths published by ONS to adjust the critical care use observed in our sample for sex and age groups (50–59, 60–69, 70–79, 80–89 and >90) [13].

### Statistical analysis

We used logistic regression and Kaplan–Meier survival plots to investigate 1-year survival following a CCA and linear regressions for trend analyses. All analyses were performed using STATA 15 software [14].

## Results

**Figure 1:**
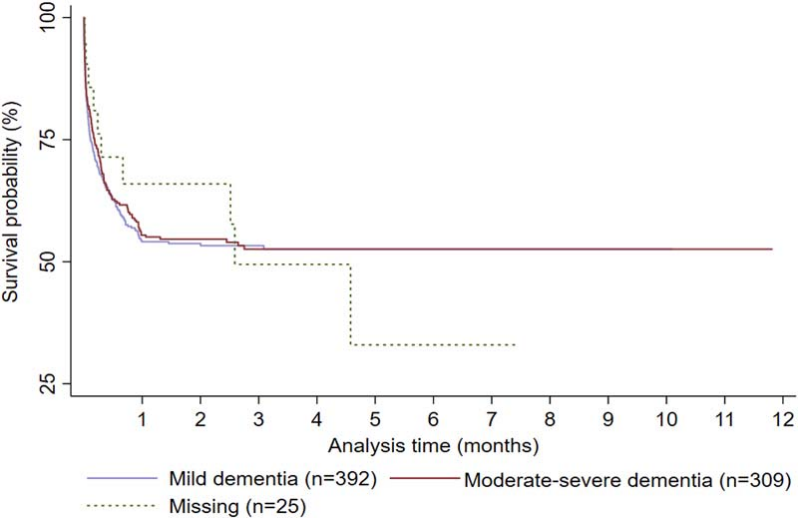
Kaplan–Meier survival plot showing the 1-year survival following a CCA in people with a previous dementia diagnosis grouped by dementia severity.

**Figure 2:**
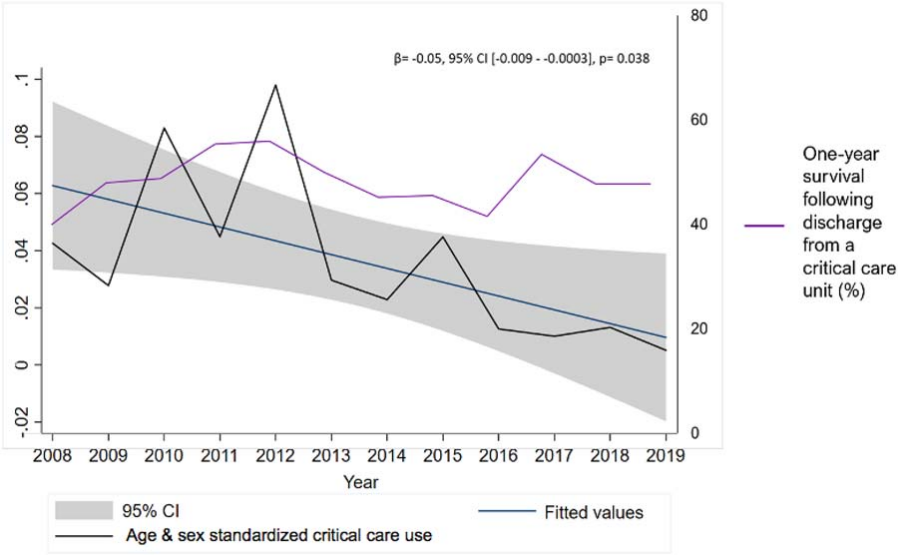
Annual age–sex-standardised critical care use among people with dementia between 2008 and 2019.

Of 19,787 people diagnosed with dementia, 726 (3.7%) had ≥1 CCA, equating to 0.27 CCAs per person-years. Those with a CCA were of median age 81 (interquartile range [IQR]: 75–86) on admission, nearly a quarter (*n* = 175, 24.1%) died in hospital, including 11.1% (*n* = 81) in the critical care unit; the remaining 551 (75.9%) were discharged after a median of 10 (IQR: 4–21) days of total hospitalisation. Compared to people who did not have a CCA, people who had ≥1 CCA were younger when diagnosed with dementia (median age of 79, IQR: 73–84 versus median age of 82, IQR: 77–87) and had a higher MMSE score (21 IQR: 17–24 versus 19 IQR: 15.0–23.0), indicating milder cognitive impairment at the time of their diagnosis (see Supplementary Data). The overall 1-year survival of people with dementia who had a CCA was 47.5% (*n* = 345). Dementia severity was not associated with 1-year survival following CCA (mild dementia versus moderate–severe dementia odds of 1-year mortality, OR: 0.90, 95% CI [0.66–1.22]) (Figure 1). Age-sex-standardised annual critical care use of people with dementia ranged between 0.5% (512 per 100,000 people with dementia) and 9.8% (9,797 per 100,000 people with dementia). Over the 12-year period from 2008 to 2019, there was a decrease in overall critical care use (*β* = −0.05; 95% CI = −0.01, −0.0003; *P* = 0.04), while 1-year survival remained steady (Figure 2). Age–sex-standardised annual critical care use during the last year of life ranged between 0.9 (905 per 100,000 people with dementia) and 3.9% (3,859 per 100,000 people with dementia). Between 2008 and 2019, there was an increase in CCAs in the last year of life (*β* = 0.11, 95% CI = 0.01, 0.20, *P* = 0.03).

## Discussion

In a large cohort of people with dementia, 3.7% experienced a CCA, potentially reflecting the careful selection process for admission to critical care units. Previous estimates of annual CCA rates for people older than 80 range between 3.0 and 16.5% [15]. The 1-year survival following CCA of 47.5% is slightly lower than that reported among people with dementia in England admitted to hospital (58.5%) [16]. It is consistent with the 1-year survival following a CCA reported among older people generally (30–70%) [17] and among people with cancer (40–55%) [18]. However, our observed proportion of CCAs that ended in death on a critical care unit (11.1%) was lower than in general population reports (14–20%) [19].

In our study, CCAs among people with dementia decreased over the years observed, while CCAs in the last year of life increased. Evidence on temporal CCA trends is scarce. Previous US studies of CCA trends of older people (2001–08) [20] and of people with dementia (1998–2015) [21] found no change. By contrast, US data showed increases in CCAs (2000, 6.1%–2007, 9.5%) [4] and life-sustaining treatments such as mechanical ventilation among people with advanced dementia [3]. Our findings agree with reported increases in other aspects of potentially burdensome care (e.g. Emergency Department attendance in the last months of life) [22]. Reasons for up-trending CCAs in the last year of life among people with dementia may be complex. The increasing emphasis on timely diagnosis of dementia in England [23], social care funding implications of having a formal dementia diagnosis [24] and provision of general hospital liaison psychiatry services [25] may have changed the composition of our cohort over time, contributing to observed trends. Following, the 2015 Prime Minister’s Challenge on Dementia policy, which aimed that 66% of people with dementia in England should receive a diagnosis, an increase in this percentage was observed: from 47 to 59% between 2011 and 2015. Implementation of incentives by the National Dementia Commissioning for Quality and Innovation [26] also led to increased recognition of dementia in acute hospitals [27]. While recognition of dementia by clinical teams has increased, skills and knowledge to deliver care for those who may be approaching the end of life are often lacking. Quality indicators such as the percentage of people with dementia who had a CCA in the last month and year of life and the documentation of treatment and care preferences may drive improvements in the care quality for people with dementia until the end of their lives [28].

This study has limitations. Our sample were diagnosed with dementia in a single mental health trust and may not be representative [29]. Furthermore, we did not have sufficient information about reasons for the CCAs, treatments received and frailty measures (e.g. the Clinical Frailty Scale) [30], which inform clinical decisions [31, 32] and have an increasingly large influence on CCA decisions than dementia severity. Finally, decisions to admit to critical care units may depend on the critical care capacity. However, data on local/regional critical care bed occupancy or capacity were not available for further exploration [33]. Study strengths include the source data linkage, which enabled us to determine CCA use among people with dementia diagnosed over a long time period. While most available information on critical care use among people with dementia is limited to those which occurred in the last year of life or only to people who had advanced dementia [3, 34], we were able to identify any CCA after the dementia diagnosis date.

Financial and individual burdens associated with dementia care are high and will increase in future. Critical care use among people with dementia and relevant outcomes, such as their survival and the concordance of care with their preferences, should be monitored to minimise burden and to meet care needs appropriately. To make good judgements about the appropriateness of care and inform service provision at the population level, access to high-quality and clinically relevant routinely collected data is essential.

## Supplementary Material

aa-22-2072-File002_afad157

## Data Availability

The datasets used in this study are based on patient data, which are not publicly available. Although the data are pseudonymized, that is, personal details of the patient are removed, the data still contain information that could be used to identify a patient. Access to these data requires a formal application to the CRIS Patient Data Oversight Committee of the NIHR Biomedical Research Centre. On request and after suitable arrangements are put in place, the data and statistical analyses used in this study can be viewed within the secure system firewall. The corresponding author can provide more information about the process.
