# Supplementary material for: Survival and critical care use among people with dementia in a large English cohort
Source: Age Ageing. 2023 Sep 8;52(9):afad157. doi: 10.1093/ageing/afad157 (PMC10484725; doi:10.1093/ageing/afad157)
Supplement: aa-22-2072-File002_afad157 [file aa-22-2072-file002_afad157.docx]

Supplementary data for the manuscript titled “*Survival and critical care use among people with dementia in a large English cohort*”

Table 1. Population characteristics

|  | Total | No critical care admission | Had ≥1 critical care admission |
| --- | --- | --- | --- |
| N | 19,787 | 19,061 | 726 |
| Sex |  |  |  |
| female | 12071 (61.0%) | 11696 (61.4%) | 375 (51.7%) |
| male | 7716 (39.0%) | 7365 (38.6%) | 351 (48.3%) |
| Age at diagnosis |  |  |  |
| <60 | 309 (1.6%) | 285 (1.5%) | 24 (3.3%) |
| >=60 - <=64 | 430 (2.2%) | 401 (2.1%) | 29 (4.0%) |
| >=65 - <=69 | 916 (4.6%) | 845 (4.4%) | 71 (9.8%) |
| >=70 - <=74 | 1998 (10.1%) | 1896 (9.9%) | 102 (14.1%) |
| >=75 - <=79 | 3615 (18.3%) | 3441 (18.1%) | 174 (24.0%) |
| >=80 - <=84 | 4820 (24.4%) | 4653 (24.4%) | 167 (23.0%) |
| >=85 - <=89 | 4749 (24.0%) | 4639 (24.3%) | 110 (15.2%) |
| >=90 - <=94 | 2278 (11.5%) | 2237 (11.7%) | 41 (5.6%) |
| >=95 | 672 (3.4%) | 664 (3.5%) | 8 (1.1%) |
| Age at diagnosis, median (IQR) | 82.0 (77.0, 87.0) | 82.0 (77.0, 87.0) | 79.0 (73.0, 84.0) |
| MMSE near diagnosis, median (IQR) | 20.0 (15.0, 23.0) | 19.0 (15.0, 23.0) | 21.0 (17.0, 24.0) |
| MMSE categories |  |  |  |
| mild | 5313 (26.8%) | 5046 (26.5%) | 267 (36.8%) |
| moderate | 4274 (21.6%) | 4115 (21.6%) | 159 (21.9%) |
| severe | 1041 (5.3%) | 1020 (5.4%) | 21 (2.9%) |
| missing | 9159 (46.3%) | 8880 (46.6%) | 279 (38.4%) |
| HoNOS moderate to severe cognitive problems | 7,830 (39.6%) | 7,593 (39.8%) | 237 (32.7%) |

MMSE: mini-mental state examination; HoNOS (health of the nation outcome scale) scores were dichotomised mild=0-2; moderate-severe=3-4) to facilitate interpretation.
